# Nurse education on sedation management in ECMO: A small-group training approach

**DOI:** 10.1177/17504589261425044

**Published:** 2026-03-10

**Authors:** Lajos Szentgyorgyi, Sam Raaj, Rebecca Billington, Sara Goddard, Samuel Henry Howitt, Miguel Garcia, Bhuvaneswari Krishnamoorthy

**Affiliations:** 1 School of Health and Society, The University of Salford, Salford, UK; 2 Manchester University NHS Foundation Trust, Cardiothoracic Critical Care Unit, Wythenshawe Hospital, Manchester, UK; 3 Imperial College London, School of Medicine, London, UK; 4 The University of Manchester, Division of Cardiovascular Sciences, Manchester, UK

**Keywords:** Small-group teaching, ECMO, Sedation, Bispectral Index, Nursing

## Abstract

Sedation during Extracorporeal Membrane Oxygenation (ECMO) is challenging due to altered pharmacokinetics. At Manchester University NHS Foundation Trust, Wythenshawe Hospital, the Richmond Agitation-Sedation Scale is the standard tool for sedation monitoring; however, it lacks precision in assessing deep sedation. Bispectral Index (BIS) monitoring offers an objective assessment but remains underused, partly because of limited nursing training. This article presents the implementation of small-group teaching sessions (SGTS) on ECMO sedation and BIS monitoring, integrated into existing ECMO refresher training. The sessions included interactive presentations, followed by anonymous feedback collection to evaluate perceptions of the clarity, relevance, appropriateness and usefulness of the SGTS. Sixty-one staff members attended, and 44 provided feedback. All respondents indicated that their learning needs were met, and 93% reported confidence in using BIS independently. The training was highly rated for clarity, relevance and practical value. Overall, our SGTS approach improved nurses’ understanding and confidence in BIS monitoring and ECMO sedation, supporting broader clinical adoption and highlighting the value of continued professional education.

## Introduction

Sedation and analgesia are fundamental to critical care, aiming to maintain comfort, relieve pain, minimise anxiety and promote a safe environment while preventing delirium (Delavari & Lak 2025, Devlin et al 2018, Freeman et al 2020, Vincent et al 2016). Although postoperative pain management is essential, the intensity and occurrence of pain in medical patients can equal that experienced after surgery or trauma (Chanques et al 2007). This is particularly true for patients receiving Extracorporeal Membrane Oxygenation (ECMO), who are often septic and critically unwell and may require multi-organ support and continuous life-saving extracorporeal circulation (deBacker et al 2018, Dzierba et al 2019, Wu et al 2021). Deep sedation is typically required during the acute phase, invasive procedures and transfers; however, once stabilised, light sedation with daily interruptions is recommended (Devlin et al 2018, Heinsar et al 2025, Szentgyorgyi et al 2025). It is widely agreed that unnecessary deep sedation delays extubation and increases morbidity and mortality (Barker et al 2024, Jaworska et al 2025, Porhomayon et al 2015, Shehabi et al 2013). Managing sedation in ECMO is particularly challenging due to altered pharmacokinetics, pharmacodynamics and the unique demands of extracorporeal circulation (Bertin et al 2025, Patel et al 2023, Szentgyorgyi et al 2025, Tukacs 2018).

While medical staff prescribe sedatives, typically nurses titrate analgo-sedation according to local guidelines, which vary considerably between institutions (Buscher et al 2013, Dzierba et al 2019, Marhong et al 2017, Reschreiter et al 2008). Sedation monitoring commonly relies on validated scores such as the Richmond Agitation-Sedation Scale (RASS), the Ramsey Sedation Scale (RSS) and the Riker Sedation-Agitation Scale (SAS), along with occasionally used local scores (Buscher et al 2013, Marhong et al 2017, Reschreiter et al 2008). However, these scales are observer-dependent, lack reliability in profound sedation and are invalid when neuromuscular blockade is used (Szentgyorgyi et al 2025). The scores do not directly measure the depth of sedation; instead, they estimate responses to stimuli that could influence the intended sedation depth (Szentgyorgyi et al 2025).

However, processed ElectroEncephaloGraphy (pEEG) technologies enable objective sedation monitoring by capturing quantifiable signals of the brain’s electrical activity, which are then converted into numerical values representing different sedation depths (Heavner et al 2022, Huespe et al 2024, Jaworska et al 2025, Kaye et al 2024, Wang et al 2017).

One of the most popular and studied pEEG-based devices, the Bispectral Index (BIS) monitor (Devlin et al 2018, Heavner et al 2022, Huespe et al 2024, Kaye et al 2024), serves as the standard pEEG-based monitor at Wythenshawe Hospital, part of Manchester University NHS Foundation Trust. However, BIS is mainly used in operating theatres for anaesthesia monitoring. Its uptake in the hospital’s critical care units has been limited by equipment availability, inadequate nursing training and low confidence in its clinical use. Consequently, the RASS has remained the standard sedation assessment tool in the hospital’s cardiothoracic critical care unit, which manages ECMO patients.

Small-group teaching sessions (SGTS) were designed, implemented and evaluated to improve staff understanding of ECMO sedation and BIS monitoring before wider implementation. The initiative also introduced a departmental PhD research project on BIS monitoring during ECMO.

The initial development of the ECMO sedation teaching package involved identifying key learning topics, including sedation principles, the new departmental sedation protocol and the introduction of BIS/pEEG monitoring. These topics were agreed upon with the lead ECMO nurse, practice-based nurse educators and the multidisciplinary ECMO team. The draft teaching package was subsequently reviewed and quality-assured by the departmental practice-based educators. However, before broad implementation, it was essential to evaluate the quality of teaching and the perceived educational value of the SGTSs used to deliver this material. Therefore, this study aimed to evaluate nurses’ perceptions of the clarity, relevance, usefulness and overall acceptability of the SGTSs.

The specific process-focused educational objectives were as follows:

1. To evaluate nurses’ perceptions of whether the SGTSs met their learning needs regarding ECMO sedation and BIS monitoring.
2. To determine whether the mode, structure and duration of the SGTSs were appropriate and conducive to learning.
3. To assess nurses’ views on the clarity, relevance and usefulness of the teaching content.
4. To gather feedback on perceived effectiveness and quality of the teaching delivery for further refinement.

Educational evaluations in nursing and clinical practice often rely on participants’ perceptions of teaching quality, clarity, relevance and usefulness when programmes are newly introduced or undergoing refinement. Measures such as satisfaction, perceived usefulness and self-confidence are commonly used as early indicators of educational effectiveness, particularly in pilot or service-evaluation contexts (Imanipour et al 2023, Kim & Im 2024, Moreno-Cámara et al 2024). Studies of simulation-based and mobile-learning teaching similarly show that perceived competence, self-efficacy and confidence offer valuable insights into whether an intervention is acceptable and appropriate before formal knowledge testing is undertaken (Fung et al 2021, Jallad 2025, Mammen et al 2025). Reviews of nursing education interventions further support this approach, noting that self-reported learning and perceived competence are widely used outcomes in early evaluations (Kulju et al 2024, Portela Dos Santos et al 2022, Woo & Cui 2025). In line with these established practices, this study evaluated nurses’ perceptions of the clarity, relevance and perceived usefulness of the STGSs.

The focus on participant feedback, rather than knowledge assessment, was intended to quality-assure the teaching package before its wider, continuous implementation across the department. An objective assessment of knowledge acquisition was beyond the scope of this initial assessment, but could be incorporated into future studies; therefore, learning objectives related to knowledge gain were not formally evaluated.

## Methods

### Staff training on ECMO sedation and BIS monitoring

Three structured teaching approaches were developed to address diverse learning needs: small-group teaching, one-to-one sessions and bedside practical sessions. McKimm and Morris describe a ‘small group’ not only by size but also by its teaching and learning context and the teacher’s facilitative role (McKimm & Morris 2009). Consequently, ‘small-group’ can be broadly defined, ranging from groups of 25 to 30 participants to one-to-one sessions. This inclusive approach accommodates shift work and varying learning styles, ensuring flexibility and accessibility (McKimm & Morris 2009, Mitchell et al 2024).

At Wythenshawe Hospital, ECMO nurses attend mandatory educational refresher days at least annually, typically twice yearly, with five to eight specialist nurses per course. SGTSs were incorporated into these sessions to enhance knowledge of ECMO analgo-sedation and develop skills for independent BIS monitoring, thereby improving sedation management. Sessions, led by a consultant anaesthetist specialising in critical care and ECMO, lasted approximately 45 min and covered ECMO sedation principles, BIS applications and targeted sedation levels.

The comprehensive interactive sessions combined didactic lectures and case-based discussions, addressing critical care sedation principles, side effects, complications and ECMO-specific pharmacokinetic and pharmacodynamic considerations. The hospital’s new ECMO sedation protocol was introduced, outlining a stepwise approach to the key characteristics of primary sedatives, analgesics and neuromuscular blocking agents. Delirium management was briefly reviewed. Sedation assessment scores and their limitations were discussed, followed by an introduction to pEEG monitoring principles, advantages and troubleshooting. Finally, the departmental ‘PRO-BIS on ECMO’ feasibility study was outlined, covering its objectives, methodology and practical implementation. Participants were encouraged to ask questions, and their feedback was used to refine subsequent sessions.

The trainer provided bedside practice updates and maintained regular verbal and online communication with ECMO nurses. A two-page laminated BIS monitoring guide was supplied for staff reference. General critical care nurses who were not part of the SGTSs could receive brief bedside instruction from trained ECMO nurses.

#### Rationale for small-group teaching

The trainer adapted small-group teaching frameworks by Burgess et al and van Diggele et al to design the programme (Burgess et al 2020, van Diggele et al 2020). Acting as a facilitator rather than a traditional, didactic lecturer, the trainer encouraged open discussion, inclusion and a reflective learning culture. Group size was limited to five to eight participants for optimal interaction. Self-reflection and feedback were used to improve subsequent sessions, with feedback serving as a key element of educator self-assessment (Burgess et al 2020). The programme followed van Diggele et al’s ‘Outcomes–Activity–Summary’ (OAS) model: identifying learning *outcomes*, structuring materials and *activities* and *summarising* with post-session feedback (van Diggele et al 2020). The sessions followed five steps: profiling the audience, defining learning outcomes, designing content, formulating assessment questions and summarising key messages (van Diggele et al 2020). A detailed description of this framework exceeds the scope of this article.

### Participants

Participants included ECMO coordinators, ECMO specialist nurses and Advanced Critical Care Practitioners (ACCPs), most of whom had limited experience with pEEG. Updates on sedation principles supported continuing professional development and promoted safe clinical practice.

ECMO patients require continuous Level 3 critical care, as defined by the Intensive Care Society and the Faculty of Intensive Care Medicine (2022). In addition to 1:1 nursing, each ECMO patient is supported by an ECMO specialist nurse, with a coordinator leading the team and doctors providing cover under the supervision of an ECMO consultant. When patient numbers are high, one specialist nurse may oversee two patients.

ACCPs can prescribe sedatives and analgesics in accordance with the departmental ECMO sedation protocol, establish vascular access and assist with clinical and sedation assessments. They also provide clinical support and are often the primary contact for nurses.

General critical care nurses were not included in this teaching due to large staff numbers at Wythenshawe Hospital’s cardiothoracic critical care unit (over 240), their high turnover, limited teaching hours and the small feasibility study sample. Lead educators agreed that one-to-one classroom sessions were impractical. Instead, a concise five-slide presentation on BIS monitoring was circulated by email to all critical care nurses. ECMO nurses, present at every ECMO bedside, supported ongoing practice-based teaching.

Anaesthetists and critical care doctors did not require additional training in sedation or pEEG monitoring, as both are included in their curricula and are regularly used in theatres.

### Staff feedback

We conducted a prospective, single-centre, cross-sectional questionnaire-based evaluation following a series of SGTS. Immediately after each teaching session, participants completed an anonymous, self-administered questionnaire [Kirkpatrick Level 1: reaction] (Allen et al 2022, Khurshid et al 2022, Kirkpatrick & Kirkpatrick 2016, Liu et al 2025, Reio et al 2017), either on paper or online. The online version, hosted on a secure NHS Microsoft Forms platform, was distributed to staff via NHS email, with emphasis on voluntary participation and anonymity. No identifiable data were collected. Completion implied consent for analysis and publication, as explained in accompanying information.

Three experts reviewed the questionnaire for content accuracy before use. These experts included a departmental practice-based ECMO specialist educator, a professor of nursing with extensive experience in nursing education and an intensive care ECMO consultant physician. It contained six closed-ended items: one on staff grade, three ‘yes/no’ dichotomous questions evaluating satisfaction with the teaching methods, learning needs and confidence in BIS use, two Likert-type-scale questions (1 = *strongly disagree* to 5 = *strongly agree*) on session relevance and quality, and one open-ended comment section (Figure 1, A–F).

**Figure 1:**
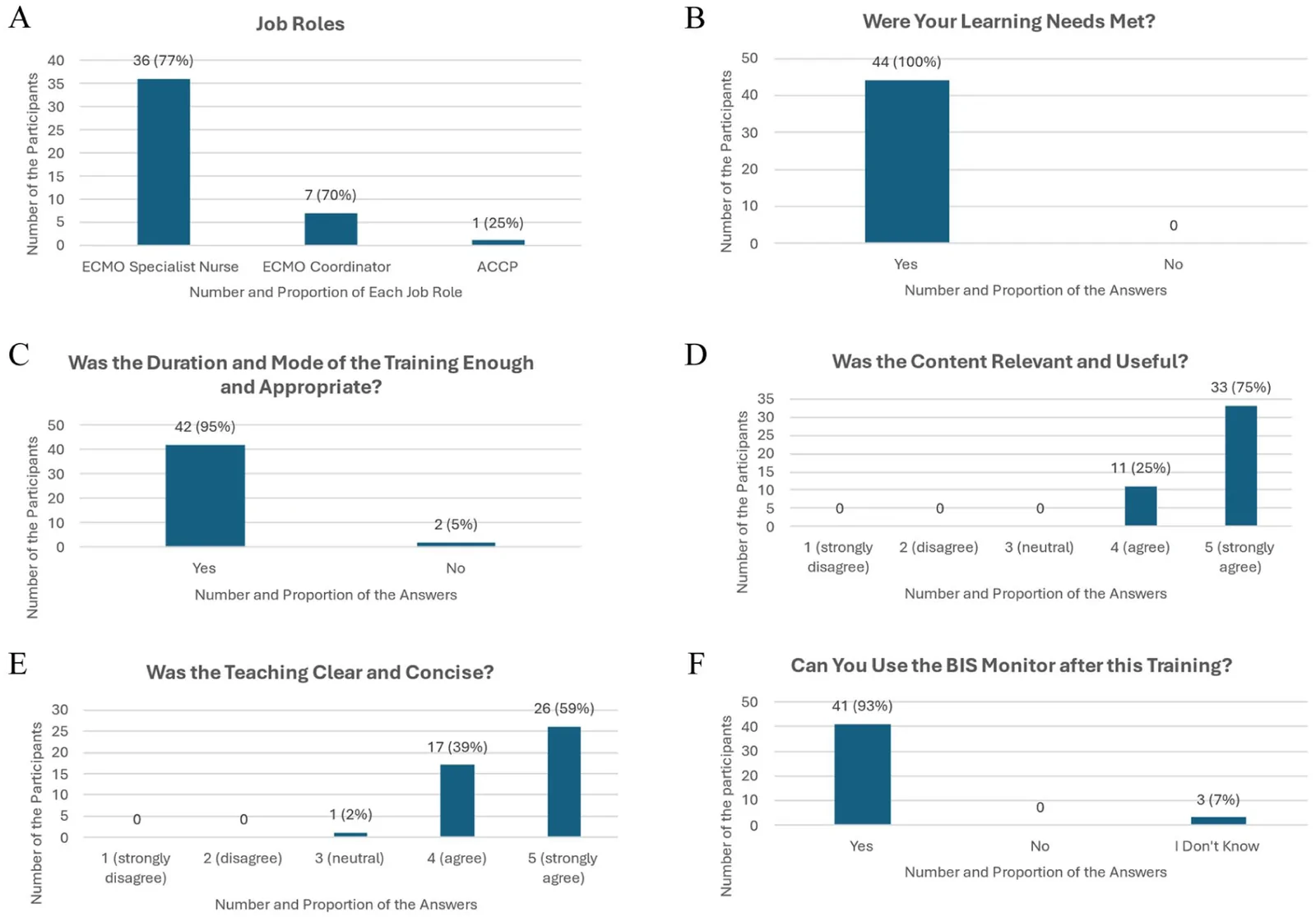
(a) Roles, numbers and proportion of staff who provided feedback after teaching. (b) Satisfaction with the fulfilment of learning needs. (c) Satisfaction with the duration and method of training. (d) The relevance and usefulness of the teaching sessions. (e) The clarity and conciseness of the teaching sessions. (f) Confidence in utilising the BIS monitor following the teaching sessions. (ECMO: Extracorporeal Membrane Oxygenation; ACCP: Advanced Critical Care Practitioner.)

A word cloud was generated to provide a quick visual overview of the most frequently used words in the feedback. This approach highlights the aspects of teaching that learners mentioned most often, enabling common impressions to be seen instantly without the need for predefined themes. A word cloud offers a fast, non-analytical visual summary of the main topics in the text. What is prominent is determined by the feedback itself, with more repeated words showing greater importance. It helps reveal what participants were trying to express, not just how often words appeared. It emphasises the intended message rather than raw frequency counts (Bletzer 2015). A word cloud does not impose categories or interpret data; it simply shows what people mention most. Using word clouds to analyse comments about staff education can help identify which topics should be prioritised for teaching and research (Mowforth et al 2023, Sellars et al 2018). When word clouds are used thoughtfully and consistently across different courses, they can increase engagement and support the development of critical thinking (deNoyelles & Reyes-Foster 2015).

#### Ethics approval

The teaching sessions and feedback analysis formed part of the ‘PRO-BIS on ECMO’ feasibility study (ISRCTN79335747; https://doi.org/10.1186/ISRCTN79335747), a postgraduate research project at the University of Salford, United Kingdom. Ethical approval was obtained from the University of Salford (HSR2223-001) and the UK Health Research Authority via the North West – Greater Manchester South Research Ethics Committee (reference 22/NW/0316; Integrated Research Application System reference 305985).

#### Data analysis

Quantitative data were summarised descriptively (median and interquartile range for ordinal data; frequencies and percentages for categorical data). Qualitative feedback comments were analysed inductively using conventional content analysis as described by Elo and Kyngas (Elo & Kyngäs 2008, Hsieh & Shannon 2005, Kuckartz & Rädiker 2023). Two investigators independently reviewed the written feedback. Owing to brevity, comments were classified as positive or negative without formal coding. Discrepancies were resolved by discussion, and responses were collated into a narrative summary.

## Results

Sixty-one staff members participated in a year-long SGTS project. Forty-four staff members (72%) provided feedback, which represented 77% of ECMO specialist nurses (36/47), 70% of ECMO coordinators (7/10) and 25% of ACCPs (1/4) (Figure 1/A).

The results are presented according to the process-focused educational objectives.

1. Learning needs assessment.

All respondents indicated that their learning needs were addressed.

2. Mode, structure and duration of the SGTSs.

Ninty-five percent (42/44) of the respondents considered both the duration and mode of training to be sufficient (Figures 1/B and 1/C).

3. Clarity, relevance and usefulness of the teaching content.

Using a 5-point Likert-type scale (1 = ‘*strongly disagree*’, 2 = ‘*disagree*’, 3 = ‘*neutral*’, 4 = ‘*agree*’ and 5 = ‘*strongly agree*’), participants rated the content as highly relevant and useful, finding the teaching to be clear and concise (Figure 1/D and 1/E). The results from the 5-point Likert-type scale (*n* = 44) indicate the following: for the question, ‘*Was the content relevant and useful?*’, respondents reported a median score of 5.0, with an interquartile range (IQR) of 0.25 and scores ranging from 4 to 5. Similarly, for the question ‘*Was the teaching clear and concise?*’, the median score was also 5.0; however, the IQR was broader, at 1.0, with scores ranging from 3 to 5.

Following the training, 93% (41/44) stated that they could use BIS monitoring independently, 7% (3/44) were uncertain, and none reported that they could not use it (Figure 1/F).

4. Feedback on perceived effectiveness and quality of the teaching delivery.

Fifteen participants (34%) submitted overwhelmingly positive written feedback, with five calling the training ‘*useful*’. Table 1 shows the comments as received.

**Table 1:** The staff’s unaltered written feedback

*1. ‘Would like to use more in practice’*
*2. ‘good presentation’*
*3. ‘very useful* *+* *interesting’*
*4. ‘Training was efficient and concise.* *I feel confident now that I will be able to use the equipment effectively’.*
*5. ‘Useful and informative presentation which was easy to understand.* *Good explanations of BIS, target parameters and overall purpose of the study’*
*6. ‘clear aims of the presentation* *I would like a refresher once we recruit the first patient’*
*7. ‘Very informative thank you* *Very clear thank you* *Learned about bis* *Yes I feel like I will be able to use’*
*8. ‘Thank you!’*
*9. ‘Good inclusion of pharmacology of sedation, all content related to patient prognosis and their quality of life. Good to get snapshot of how knowledgeable our consultant colleagues are. Haven’t yet had to use it. Although the talk included lots of photos of the BIS, would maybe be useful to have a machine at the talk when thinking about the interface, simple stuff like what the different buttons do and how to troubleshoot’.*
*10. ‘I have used it’*
*11. ‘A very useful study’*
*12. ‘The slides were a bit busy but the information was useful.* *BiS monitoring sounds like a useful tool for measuring sedation level. I’m interested to start using it in practice. Interesting presentation, thanks’.*
*13. ‘I would prefer more in depth training. Would prefer print out version as well as the slide. I would prefer a longer session* *+* *scenario questions* *+* *more in depth learning.* *I would need further training’.*
*14. ‘Each day, clinical scenarios are different. Thank you so much for your lecture. I would appreciate if you could teach us more at bedside. As you said, sedation weaning is variable depends upon patient’s condition so it would be appreciated if we get expert’s advice/knowledge sharing at bed side’.*
*15. ‘Learnt how to escalate or decrease sedations clearly. Overall the session was a good refresher and informative’.*

Figure 2 shows a word cloud of common feedback words, generated with https://www.wordclouds.com/.

**Figure 2:**
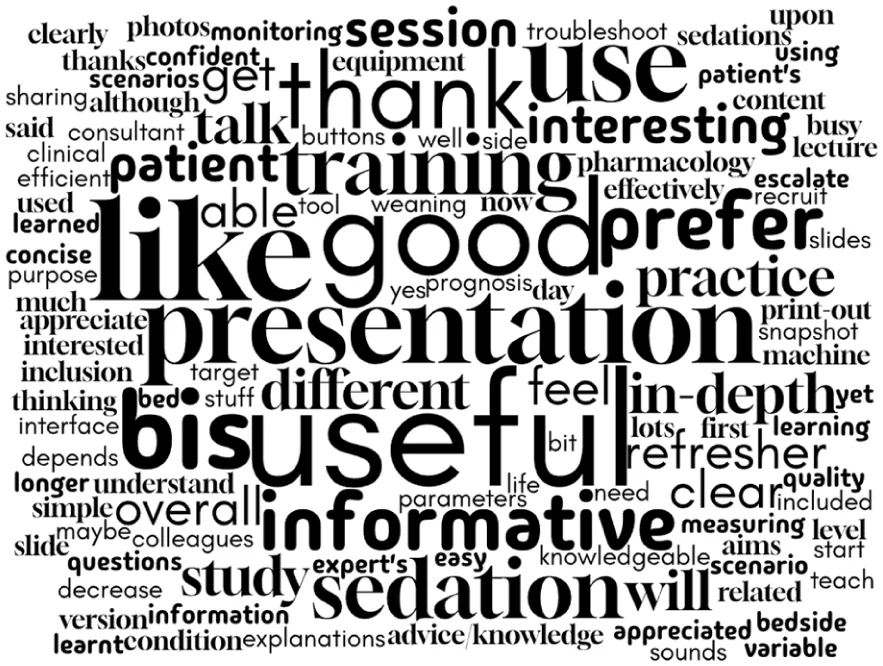
Word cloud of teaching feedback

## Discussion

### The benefits of SGTS

Although BIS monitoring is established in many critical care units, the ECMO staff at Wythenshawe Hospital have limited experience using it. Its infrequent application is linked to the absence of specific guidance and formal training. Therefore, a year-long SGTS programme involving 61 staff members was undertaken to improve knowledge of sedation, ECMO-specific challenges, the departmental protocol and BIS monitoring. The sessions also introduced a feasibility study evaluating BIS monitoring during ECMO, designed to inform a future clinical trial and routine implementation within the unit.

Educating nursing colleagues was highly beneficial, as it used real-life scenarios to clarify misunderstandings about ECMO analgo-sedation, sedation monitoring and BIS monitors. The participants included those directly responsible for the care of ECMO patients. Regular refresher days provided a suitable setting for interactive teaching, which received consistently positive feedback. The sessions improved understanding of ECMO sedation and monitoring, clarified misconceptions, strengthened BIS skills and promoted bedside education among general critical care nurses.

Health Education England highlights education, training and development as vital for patient safety, emphasising it is everyone’s shared responsibility within the healthcare system (Health Education England 2019, Maddison 2024). Interactive small-group education improves confidence and facilitates evidence-based practice, particularly when delivered by experienced clinicians (Portela Dos Santos et al 2022, Sapri et al 2022). Integrating SGTSs within the hospital’s ECMO refresher programme supports institutional learning, improves care, enhances motivation and confidence and provides opportunities to implement new technologies and conduct research (Lyman et al 2019, 2020).

Our team-based small-group sessions focused on active participation, task-oriented work (such as ECMO sedation practice and pEEG monitoring) and reflection, following the principles outlined by R. W. Jones (2007). This format encouraged self-motivation and learner satisfaction, enabling participants and educators to check understanding in real time. Frequent opportunities to identify knowledge gaps and ask questions promoted deeper learning and interactivity. The method fostered team spirit, strengthened collegiality and gave the group collective ownership of learning, resulting in teaching outcomes consistent with those described by Jones (2007).

From a research perspective, effective multidisciplinary training was essential before launching the departmental BIS study, which depended on nursing involvement (Black et al 2019, Ferreira et al 2022, Health Research Authority 2020, Health Research Authority, Medicines and Health care products Regulatory Agency 2017). Standard Good Clinical Practice (GCP) training modules (Bhatt 2023, International Council for Harmonisation of Technical Requirements for Pharmaceuticals for Human Use, 2023, World Health Organization 2005, 2024) alone may not sufficiently prepare inexperienced staff, and they are impractical for mandatory rollout to all clinical personnel, as suggested by Bechtel et al (2020). A tailored, multi-method teaching approach may have proven more effective than a didactic, one-size-fits-all approach for training clinical staff and research personnel, as well as for verifying their qualifications. This strategy combines GCP principles with role- and protocol-specific practical instruction, enhancing research quality, participant protection and data integrity (Bechtel et al 2020, Bhatt 2023, World Health Organization 2024). Furthermore, it may also help attract and retain staff from underrepresented groups, as was highlighted by Niranjan et al (2019). This issue may be particularly significant in a multicultural, diverse city such as Manchester, United Kingdom.

Our results show that participants recognised key learning topics – including sedation principles, the new departmental sedation protocol and BIS/pEEG monitoring principles – as being appropriately addressed. In addition, the approach achieved the process-focused educational objectives: evaluating nurses’ perceptions of whether the SGTSs met their learning needs regarding ECMO sedation and BIS monitoring; assessing the suitability of the mode, structure and duration; examining views on the clarity, relevance and usefulness of the content; and gathering feedback on the effectiveness and quality of delivery to guide future improvements.

### The value of feedback after SGTS

Using participant feedback, before or instead of formal knowledge testing, is common in nursing and clinical education. Studies have shown that satisfaction, perceived confidence, self-efficacy and perceived competence are valid early indicators of educational quality and acceptability, particularly during the initial implementation of new teaching materials (Jallad 2025, Kim & Im 2024, Moreno-Cámara et al 2024). Several evaluations have relied on self-reported outcomes to refine teaching packages, including simulation-based education, feedback-focused training and structured skills development (Imanipour et al 2023, Mammen et al 2025, McGinness et al 2020). Reviews also highlight that perceived usefulness, competence and self-reported learning are widely used as legitimate outcomes in early educational assessments (Fung et al 2021, Kulju et al 2024, Portela Dos Santos et al 2022, Woo & Cui 2025). Within this context, the present evaluation appropriately focused on nurses’ perceptions of clarity, relevance and effectiveness as part of a preliminary assessment of a new departmental teaching package.

Giving and receiving feedback is vital for personal and professional development. It encourages reflective learning and, with effective communication, creates a safe, supportive learning and working environment (Peters et al 2023). Asking for feedback also strengthens trainers’ self-reflection (Burgess et al 2020). The SGTS evaluation used a prospective, cross-sectional, mixed-methods design, achieving a 72% response rate – higher than expected – although ACCPs responded less frequently.

Feedback indicated that participants found the session length and format appropriate, and that the learning objectives were met. Most agreed strongly that the content was relevant, useful and clearly presented. After training, 93% of participants expressed confidence in using BIS independently, and 7% remained uncertain. It is worth noting that some nurses had previous experience with this technology.

Only one-third submitted written comments, which were overwhelmingly positive, and several requested further training. Overall, the reception and feedback exceeded expectations, highlighting the effectiveness and relevance of the educational approach.

The word cloud reflects the tone of the comments, with prominent terms such as ‘*like*’, ‘*presentation*’, ‘*useful*’, ‘*good*’ and ‘*informative*’ indicating that the teaching was generally well-received and considered valuable. Repeated references to *‘training*’, *‘BIS*’, *‘sedation’* and *‘use’* suggest that the clinical focus of the teaching was well aligned with learners’ needs, and the practical and clinical aspects of the session were appreciated. Other noticeable words, including *‘interesting*’, *‘session*’, *‘prefer*’, *‘practice*’, *‘different*’, *‘in-depth*’, *‘study*’, *‘will*’, *‘patient*’ and *‘talk*’, suggest that participants engaged with the material and considered how it related to their work or future learning. These comments may indicate that participants feel more *‘confident’* and *‘able’* to use the equipment, while others express interest in *‘practice’*-based or more *‘in-depth’* teaching, including *‘bedside’* demonstrations and scenario-based discussions. Words such as *‘refresher’* and *‘clear’* appear less frequently but reflect individual appreciation for the consolidation of knowledge. Overall, the feedback suggests that the session was well-received, informative and helpful, with some desire for further practical and advanced training.

### Limitations and future directions

Several limitations should be acknowledged. This single-centre study mainly involved senior nursing staff – ECMO nurses, coordinators and ACCPs – who are likely more motivated and experienced than general critical care nurses, limiting generalisability. The dual focus on ECMO sedation and BIS monitoring may also restrict applicability to broader critical care populations and may risk splitting attention between the topics. As the content evolved iteratively, later sessions might have benefited from improvements that could have resulted in minor inconsistencies.

The BIS-centred content may limit transferability to other pEEG systems. Equipment shortages restricted hands-on training, and extending the programme to all critical care nurses was impractical, leading to reliance on peer teaching.

Evaluation relied on self-reported perceptions and confidence rather than formal assessments of competency and knowledge gain, which may not reflect actual proficiency, although it still indicates preparedness.

The word cloud should also be interpreted with some caution. It reflects only how often individual words appear, not how they are used in sentences or whether they were meant positively or negatively. Different words with a similar meaning (e.g. ‘*thank*’ and ‘*appreciate*’) are counted separately, and less common but important comments may appear very small or be missed entirely. The appearance of the cloud is also influenced by technical choices, like which common words are removed beforehand. Therefore, the word cloud is best used as a simple, concise visual summary rather than a substitute for detailed qualitative analysis, such as thematic coding of the full comments (Henderson et al 2025). Nonetheless, given the few and brief comments, a word cloud was considered an appropriate and helpful visual aid for understanding participants’ general opinions of the SGTSs.

Future programmes should expand participation to include general critical care nurses and additional departments, and incorporate regular refresher courses. Adequate device availability would improve hands-on practice. Structured post-implementation feedback could refine training and content while enabling the assessment of clinical competence. In addition, future adoption of more complex evaluation models may enable more accurate assessment of acquired knowledge (Allen et al 2022).

## Conclusion

Our SGTS approach improved nurses’ perceived knowledge and confidence in managing ECMO sedation. In addition, the approach successfully achieved its process-focused educational objectives and addressed the identified learning needs. Feedback regarding the effectiveness and quality of delivery will guide future educational improvements.

These findings should be interpreted in light of the study’s limitations, including its single-centre design, participant profile and reliance on self-reported outcomes, which may limit generalisability. Nevertheless, the study highlights the importance of ongoing high-quality educational initiatives to strengthen ECMO sedation management and supports further evaluation of the SGTS approach across broader critical care populations.

## Data Availability

The authors affirm that the methods used in the data analyses are suitably applied to their data within their study design and context, and the statistical findings have been implemented and interpreted correctly. The authors agree to take responsibility for ensuring that the choice of statistical approach is appropriate and that it is conducted and interpreted correctly as a condition for submitting to the Journal. The data sets generated and analysed during the current project consist of anonymised staff feedback responses and aggregated summary statistics. Because the feedback was obtained from a small clinical team, releasing the complete raw data could risk inadvertently re-identifying individual participants. Anonymised, group-level results are included in the article. A minimally redacted data set (with free-text comments removed) and the analysis code are available from the corresponding author upon reasonable request and with Manchester University NHS Foundation Trust’s permission, aligning with local information-governance policy.
